# The Use of a Vibro-Acoustic Based Method to Determine the Composite Material Properties of a Replicate Clavicle Bone Model

**DOI:** 10.3390/jfb11040069

**Published:** 2020-09-24

**Authors:** Quentin Goossens, Sanne Vancleef, Steven Leuridan, Leonard Cezar Pastrav, Michiel Mulier, Wim Desmet, Jos Vander Sloten, Kathleen Denis

**Affiliations:** 1 Department of Mechanical Engineering, Biomechanics Section, KU Leuven, 3000 Leuven, Belgium; sanne.vancleef@kuleuven.be (S.V.); steven.leuridan@gmail.com (S.L.); Leonard.Pastrav@kuleuven.be (L.C.P.); jos.vandersloten@kuleuven.be (J.V.S.); kathleen.denis@kuleuven.be (K.D.); 2 Division of Orthopaedics, University Hospital Leuven, 3000 Leuven, Belgium; michiel.mulier@uzleuven.be; 3 Department of Mechanical Engineering, MSD Section, KU Leuven, 3000 Leuven, Belgium; wim.desmet@kuleuven.be

**Keywords:** clavicle, replicate composite bone, material parameters updating, modal analysis

## Abstract

Replicate bones are widely used as an alternative for cadaveric bones for in vitro testing. These composite bone models are more easily available and show low inter-specimen variability compared to cadaveric bone models. The combination of in vitro testing with in silico models can provide further insights in the evaluation of the mechanical behavior of orthopedic implants. An accurate numerical representation of the experimental model is important to draw meaningful conclusions from the numerical predictions. This study aims to determine the elastic material constants of a commonly used composite clavicle model by combining acoustic experimental and numerical modal analysis. The difference between the experimental and finite element (FE) predicted natural frequencies was minimized by updating the elastic material constants of the transversely isotropic cortical bone analogue that are provided by the manufacturer. The longitudinal Young’s modulus was reduced from 16.00 GPa to 12.88 GPa and the shear modulus was increased from 3.30 GPa to 4.53 GPa. These updated material properties resulted in an average natural frequency difference of 0.49% and a maximum difference of 1.73% between the FE predictions and the experimental results. The presented updated model aims to improve future research that focuses on mechanical simulations with clavicle composite bone models.

## 1. Introduction

Composite replicate bone models are often used as an alternative to cadaveric bone models for in vitro biomechanical research, for example, to assess the performance of orthopedic plates and screws used for clavicle fracture fixation [1,2,3,4,5,6]. They are made of short glass fiber-reinforced epoxy resin (the cortical bone analogue) with a polyurethane foam center (the cancellous bone analogue). These composite bone models are more easily available, easier to handle and store and show low inter-specimen variability compared to cadaveric bone models [7,8,9], which makes them attractive for biomechanical testing.

Finite element (FE) analysis has become more popular and accepted in orthopedic research as it forms a valuable alternative or addition to experimental work. FE analysis offers the possibility to analyze biomechanical structures while controlling multiple parameters, such as loading/boundary conditions, geometry, material properties etc., contributing to a better insight in the studied problem. More specifically, FE models have found their merit in clavicle-related orthopedic research, such as the assessment of stress, strain and micro-motion distribution in clavicle fracture fixation treatments [4,10,11,12,13,14,15] or the quantification of stress shielding around clavicle fracture fixation implants [16].

Validation of these FE bone models is crucial to obtain a biofidelic model and to be able to draw conclusions from FE predictions [17]. Implementing accurate material models in the FE model is one of the important elements to obtain conformity between the FE and experimental models [18]. Other important elements are a correct geometry and loading/boundary conditions of the FE model. In order to validate these models, different techniques can be used to compare the experimental model behavior with corresponding FE predictions. The experimental behavior can be characterized by strain measurements using strain gauges or digital imaging correlation techniques (DIC) under standardized tests (tensile, compression or four-point bending tests). This approach is very suitable to compare experimental strains with FE predicted strains [8,19,20,21,22,23]. However, the elastic material constants, which are essential for the FE model, are difficult to determine directly out of these tests given the rather complex geometry of the bone models.

As an alternative, experimental modal analysis can be used to validate and determine the material properties of mechanical structures [24]. Modal analysis is a non-destructive technique that characterizes the vibrational behavior of a structure by its modal parameters (natural frequencies and mode shapes). These modal parameters are determined by the global mass and stiffness distribution of the structure which allows a global validation of the entire model, unlike FE validation studies that use local strain gauge measurements [25]. The material properties of the FE model can be determined by updating these properties to minimize the difference between the FE modal parameters and the experimental modal parameters [26].

The combination of experimental and numerical modal analyses has also been used in biomechanical research to determine material properties of ex vivo bone samples [27,28] and of cadaveric and replicate whole bones [7,25,29,30], and to validate FE pelvic bone models [31,32].

This study aimed to determine the elastic material constants of the cortical bone analogue of a fourth-generation composite clavicle model using a modal analysis-based approach. As far as the authors know, this is the first study that uses a modal-based updating approach to determine the material properties of a fourth-generation clavicle composite bone model.

## 2. Materials and Methods

### 2.1. Composite Bone Model

**Figure 1:**
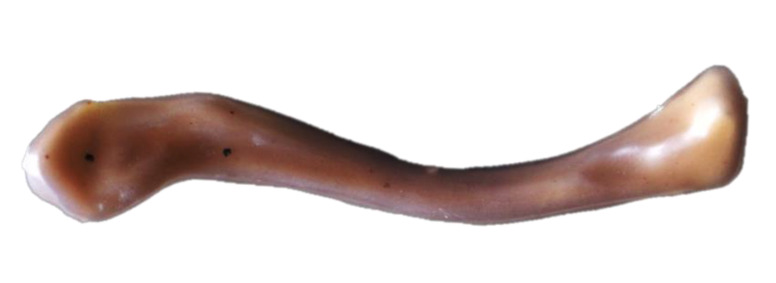
Example of a fourth-generation composite left clavicle used in this study (model no. 3408-1, Sawbones, Vashon Island, WA, USA).

A fourth-generation composite clavicle (model no. 3408-1, Sawbones, Vashon Island, WA, USA, Figure 1) was tested during this study. This fourth-generation composite bone model consists of a cortical bone substitute layer made of a glass fiber-reinforced epoxy and a cancellous bone substitute made of solid polyurethane foam. The mass of the composite bone model was measured using a precision scale (Acculab Atilon, Sartorius Group, Göttingen, Germany).

### 2.2. Finite Element Model

A computed tomography (CT) scan with a slice thickness of 0.6 mm (Somatom Force, Siemens AG, Erlangen, Germany) was made of the studied composite clavicle model to capture the geometry of the model. Based on this CT scan, a finite element model was built using 3-matic (Materialise NV, Leuven, Belgium) software. The finite element model consisted of 136,126 quadratic tetrahedral elements. Cortical and cancellous bone zones were transferred from the CT scan to the finite element model based on the gray values using Mimics (Materialise NV, Leuven, Belgium) software.

The FE model contained an isotropic material to model the cancellous bone analogue and a linear elastic transversely isotropic material to model the cortical bone analogue. A transversely isotropic material was chosen as isotropic material failed to accurately model the cortical bone analogue in previous modal FE research [7,21].

**Figure 2:**
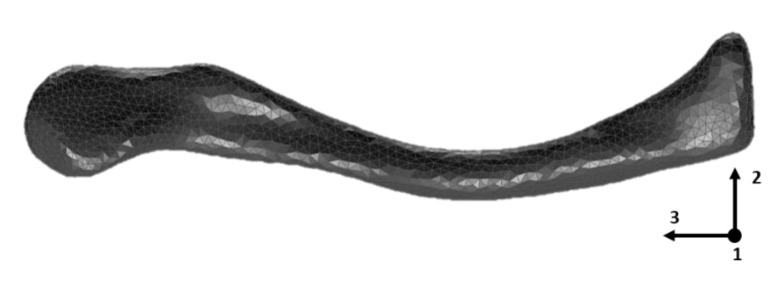
Finite element model of the composite clavicle model generated from a computed tomography (CT) scan of the tested specimen.

While the isotropic elastic material model requires two independent material properties to describe its behavior (e.g., E and ν), the transversely isotropic material model requires five independent material properties: E_11_ = E_22_, E_33_, ν_23_, ν_12_ and G_13_ = G_23_. The plane of isotropy (defined by axis 1 and axis 2 in Figure 2) of the transversely isotropic material was oriented normal to the longitudinal axis (axis of minimum moment of inertia) of the model (axis 3 in Figure 2). The initial material properties that were updated in this study are supplied by the manufacturer and are listed in Table 1.

### 2.3. Experimental Acoustic Modal Analysis

**Figure 3:**
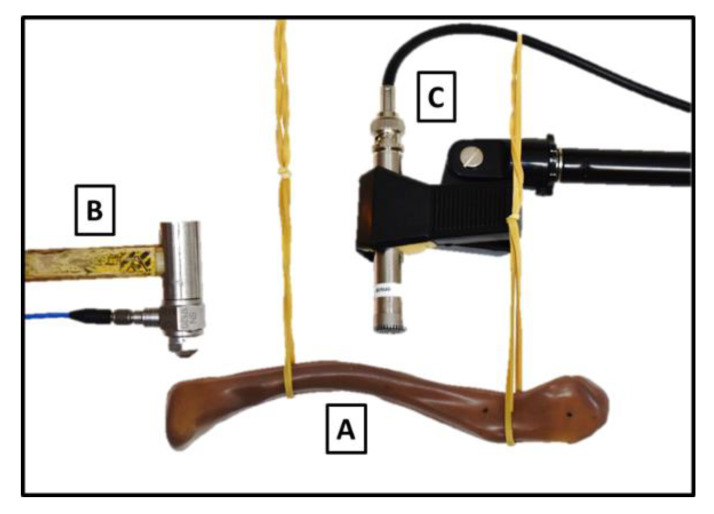
Experimental modal analysis set-up. The replicate clavicle model (**A**) was suspended using elastic bands. Impact excitation was performed with a modal hammer (**B**) perpendicular to the surface and the vibro-acoustic response was captured using a microphone (**C**).

An experimental acoustic modal analysis was done on the studied composite clavicle model. An acoustic approach was preferred over a mechanical approach (accelerometers) to prevent an imminent mass loading effect of the accelerometers on the lightweight clavicle composite model. The composite clavicle model was horizontally suspended using elastic bands to simulate free-free boundary conditions as used in the FE analysis [26]. A modal impact hammer equipped with a force sensor (Model 208C03 PCB Piezotronics, Depew, NY, USA) was used to excite the composite clavicle model at six different locations. These locations were equally distributed over the model in two perpendicular directions in order to accurately capture the modal behavior of the model. Excitations were performed perpendicular to the surface at the marked locations. A 1/2″ microphone (Model 378B02, PCB Piezotronics, Depew, NY, USA) was used to measure the vibro-acoustic response of the composite clavicle model. Figure 3 shows the experimental set-up. Five impact measurements were averaged per excitation location. The input (force) and output (pressure) signals were recorded using a spectral analyzer (LMS SCADAS III, Siemens PLM Software, Leuven, Belgium) and the corresponding modal analysis software (LMS Test. Lab, Siemens PLM Software, Leuven, Belgium) at a sample frequency of 20,480 Hz. Six frequency response functions (FRF) were obtained out of these input-output measurements. The natural frequencies were extracted out of this set of FRFs using the Polymax algorithm integrated in the Test.Lab software (LMS Test.Lab 17, Siemens PLM Software, Leuven, Belgium). The first five modes were considered in this updating study, corresponding with a frequency range from 50 to 4000 Hz.

### 2.4. FE Parameter Updating Protocol

In a first step, the densities of the cortical and cancellous bone analogue material of the FE model were updated to match the numerical mass with the experimental mass of the composite clavicle model. In a second step, the elastic material constants of the cortical and cancellous analogue material of the FE model were updated in order to minimize the error between the numerical and experimental natural frequencies using an iterative optimization procedure. Modal analysis was done on the FE model at every iteration step to determine the natural frequencies and corresponding mode shapes by applying first the initial, and subsequently, the updated material properties. MSC Patran and Nastran FE software (MSC Software, Newport Beach, CA, USA) was used to perform the numerical modal analyses. The longitudinal E-modulus (E_33_) and off-plane shear modulus (G_13_ = G_23_) of the cortical bone analogue were altered during this optimization process, and the other material properties of cortical and cancellous bone analogue were held constant. The choice for these two material properties was based on the sensitivity of the natural frequencies to the different material properties. Table 2 shows these sensitivities. According to this table, E_33_ and G_13_ = G_23_ have the biggest influence on the natural frequencies. More specifically, the tensile modulus E_33_ has the largest influence on the bending modes (1, 2, 4, 5), while the shear modulus G_13_ = G_23_ has the largest influence on the torsional mode (3). Updating these two properties will lead to a fast convergence of the minimization problem with realistic (small) alterations of the material properties. E_33_ and G_13_ = G_23_ were iteratively altered until the average and maximum error between the numerical and experimental natural frequencies converged to a minimum (average <0.5%, maximum error <2%).

## 3. Results

In order to match the mass of the numerical model with the experimental determined mass (39.1 g), the density of the cortical bone analogue material was increased from 1.640 g/cc to 1.658 g/cc and the density of the cancellous bone analogue material was kept constant at 2.700E^−1^ g/cc.

**Figure 4:**
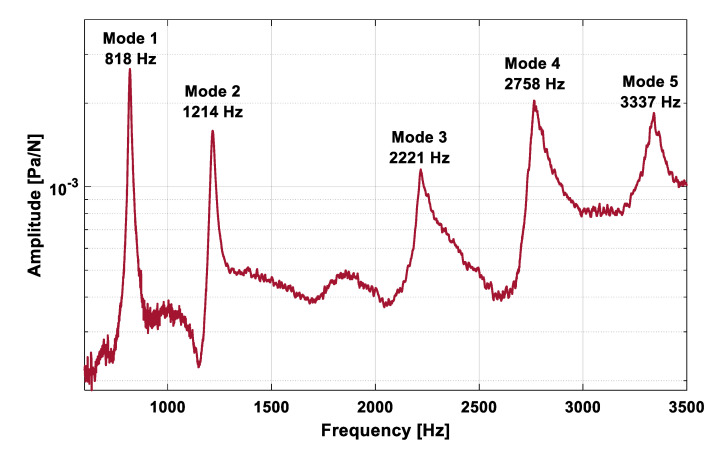
Superposition of the experimentally measured acoustic frequency response functions (FRFs) and the corresponding first five modes of the composite clavicle.

**Figure 5:**
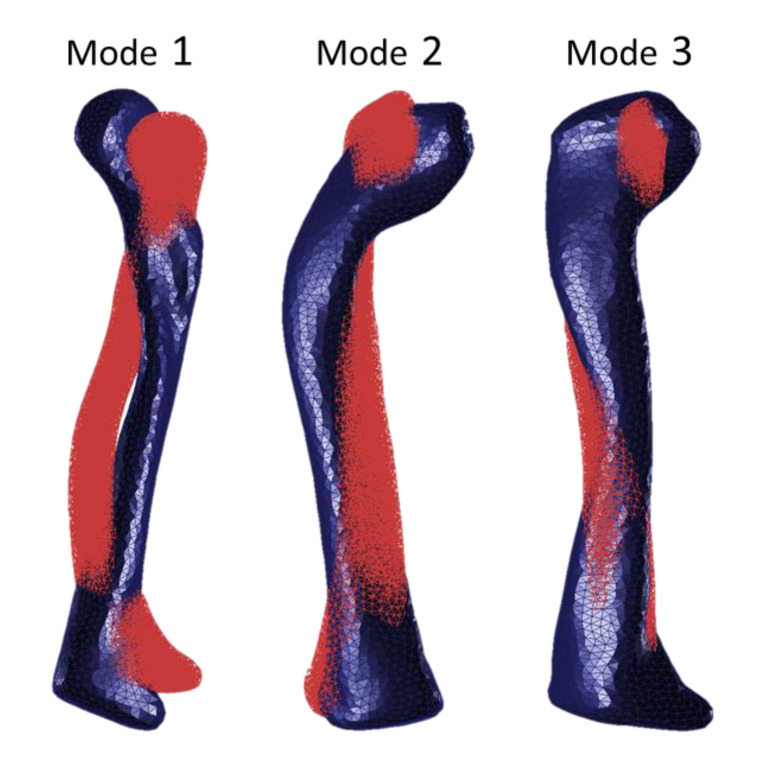
First three FE-predicted mode shapes of the clavicle model: first bending mode in the transverse plane (Mode 1), first bending mode in the coronal plane (Mode 2) and first torsional mode (Mode 3). The wireframe displays the undeformed shape.

The experimental and numerical natural frequencies (before and after updating the material properties) are shown in Table 3. Figure 4 shows the superposition of the six acoustic FRFs that were measured during the acoustic modal analysis experiment of the composite clavicle model; the first five modes are marked on this graph. Figure 5 shows the corresponding mode shapes of the first three natural frequencies of the clavicle model. The average absolute natural frequency difference before updating was 4.89% with a maximal difference of 9.57% (Mode 3) within the frequency range of interest. E_33_ was decreased from 16.00 GPa to 12.88 GPa and G_13_ = G_23_ was increased from 3.30 GPa to 4.53 GPa by the updating procedure, which resulted in an average natural frequency difference of 0.49% and maximum of 1.73% (Mode 5). An overview of the updated material properties is listed in Table 4.

## 4. Discussion

This study determined the elastic material constants of a fourth-generation clavicle composite bone (model no. 3408-1, Sawbones, Vashon Island, WA, USA). Experimental and numerical modal analyses of the composite bone model were performed. The material properties of the FE model were updated using the experimental results to ensure that the predictions of the FE model corresponded to the modal experimental behavior of the composite bone model. FE analysis is widely used as a relatively time efficient and convenient method to analyze a wide range of mechanical structures. When experimental models are used in combination with their in silico twin in order to gain further insight, it is crucial to use accurate material properties to obtain correct and comparable results [17].

The major benefit of the selected modal-based updating approach is its non-destructive nature; the updating procedure can be performed without damaging or altering the tested specimen and can be carried out at any desired point of time. This allows the user to directly obtain updated elastic material constants for the in silico twin prior to performing the intended experimental work with the in vitro twin. In contrast to alternative methods to determine the elastic material constants, such as tensile, compressive or three-point-bending tests, the tested specimen is kept intact, the testing time is very low and no additional specific loading equipment is required.

Alternative experimental FE model validation methods that are used nowadays for bone models compare and match numerical and experimental strain values. These strain values can be measured experimentally at discrete locations along the specimen using strain gages [8,22,23,35] or by measuring the strain distribution along a surface of the specimen using digital image correlation techniques (DIC) [18,20,21]. A benefit of this strain-based method is that the entire simulation procedure, encompassing mechanical properties and boundary conditions, is validated. On the other hand, the specific loading and boundary conditions can have a large influence on the results and need to be taken into account carefully as they may introduce uncertainties and so hamper the validation procedure [22]. The presented modal-based validation approach isolates the bone model from its loading and boundary conditions, which makes it possible to directly derive the elastic material constants given an accurate geometrical FE model. Moreover, the measured modal parameters are determined by the global structural properties (mass and stiffness) of the specimen, which makes it a global validation method, similar to a DIC strain validation approach. This is not the case for local strain gages-based validation as it measures the strain at a discrete number of locations.

The methodology of updating FE models with experimental vibrational data is widely used in civil engineering applications [24,36]. Besides civil engineering applications, this methodology also showed value in biomechanical applications where cadaveric bone models were studied [25,29,30,31,32,37]. A previous study by Leuridan et al. used this methodology to define the elastic material constants of different composite bone models (fourth-generation composite cylinder, tibia and femur) using modal experimental results [7]. In addition to the existing literature, this study aimed to validate a composite clavicle FE model by updating the elastic material constants using experimental modal analysis.

These updated FE models using vibro-acoustic techniques are valuable in research areas using vibrational measurement methods, such as the assessment of bone healing [38,39], the assessment of the influence of fixation [40] or shape and density distribution [41] of bone models on their natural frequencies and implant stability assessment [34,42,43,44,45].

In addition to implementing these updated models in vibration-based biomechanical research as stated above, vibration-validated clavicle FE models can also find their application in numerical orthopedic research applications, such as the assessment of clavicle fracture fixation treatments [4,10,11,12,13,14,15,46], influence of patient-specific parameters on clavicle fractures [47] or analysis of stress shielding effects in clavicle fracture fixation treatments [16].

A transversely isotropic material model was chosen in this study over an isotropic material model. Previous studies showed that FE bone models using transversely isotropic material models more closely represent the experimental modal behavior of composite bone models [7,21,48]. Isotropic material models can find their use when analyzing the global structural behavior of bones with an inhomogeneous material model. These inhomogeneous material models are used for computed tomography-obtained FE bone models where a gray values-based material assignment is used [49]. On the other hand, when the local structural behavior or small bone samples are analyzed, an anisotropic material model tends to provide more biofidelic results [50]. When working with a homogenous material model (as is the case in this study), isotropic material models tend to fail, especially when torsional behavior is studied [7].

The E_33_ Young’s modulus and G_13/23_ shear modulus of the FE model cortical bone analogue were updated during this validation study. The other elastic material constants were kept constant during the updating process as these properties showed to have limited influence on the studied natural frequencies compared to E_33_ and G_13/23_ (Table 2). Updating E_33_ and G_13/23_ resulted in a fast convergence of the minimization problem with realistic alteration of the material properties. When comparing the elastic material constants of the cortical bone analogue before and after updating, the longitudinal Young’s modulus was decreased from 16.00 GPa to 12.88 GPa (20%) and the G_13/23_ shear modulus was increased from 3.3 MPa to 4.53 MPa (37.3%). These findings are in accordance with the previous modal-based validation study of [7] that reports a lower longitudinal Young’s modulus (13.90 GPa) and higher shear modulus (3.93 GPa) of the cortical analogue material after updating, compared to the manufacturer’s provided properties. Other experimental validation studies of composite bone models with a strain based validation method have similar findings. [51] determined a Young’s modulus of 10.7 GPa out of a three-point bending test on the cortical analogue material of a fourth-generation femur model. [48] found more accurate FE simulation results of a third-generation tibia composite model when using a cortical bone analogue’s longitudinal Young’s modulus of 11.39 GPa that was derived from a theoretical composite material model. In the work of [21], higher experimental positive strains were measured in a composite bone model compared to the FE predicted strains (using a longitudinal Young’s modulus of 16.7 GPa) under a single leg stance loading regime, which is in line with the presented findings regarding the reduction of the longitudinal Young’s modulus.

It is important to mention that the accuracy of both the experimental acoustic modal analysis and the FE model are significant to obtain reliable results. Therefore, to demonstrate the validity of the methods presented in this study, a verification experiment was conducted using a well-known reference specimen. This specimen was manufactured from pure magnesium (99.5%). Consequently, the elastic material constants and mass density are known (E = 44.00 GPa, G = 17.10 GPa, ρ = 1.74 g/cc [52]). A beam shaped specimen (5 mm × 5 mm × 100 mm) was manufactured and its dimensions were accurately measured with a caliper. A corresponding FE model (21816 hexahedral elements) was built according to the same procedure presented for the clavicle model throughout this study and using the aforementioned material properties. The first six natural frequencies of the specimen (which correspond to a frequency range up to approx. 13 kHz) were determined experimentally and numerically, similarly as described in Materials and Methods. The results showed a high degree of correspondence between the experimentally and numerically determined natural frequencies (an average difference of 0.19% with a maximum of 0.92%). This verification experiment with a reference specimen demonstrates the accuracy of the methodology that compares acoustic experimental modal analysis with the numerical predictions that is presented in this study.

A limitation of this study is that only one composite clavicle bone model was tested. This was due to the requirement of the material properties of this specific clavicle specimen for further FE and experimental research. However, the inter-specimen variability in different other composite bone models of the same manufacturer made out of identical materials is reported to be very low; 1.2% up to 10% for humerus models [9,53], 0.9% up to 9.3% for tibia models [7,8,54], 0.8% up to 7.5% for femur models [7,54] and 0.3% up to 2.5% for cylinder models [7]. Given these reported findings, it can be expected that composite clavicle bone models possess similar low inter-specimen variability.

Another limitation of this study is the assumption that the plane of isotropy is oriented normal to the longitudinal axis of the model (axis of minimum moment of inertia, axis 3 on Figure 2). In most long bones (femur, tibia or humerus) the plane normal to this longitudinal axis corresponds to the anatomical plane of transverse isotropy. However, in the case of the clavicle model, which has a rather curved geometry compared to the above mentioned long bones, this plane normal to this longitudinal axis does not completely match the anatomical plane of transverse isotropy which has an orientation that corresponds to the local cross section of the bone model. As a result, this assumption could be compensated during the presented FE updating procedure by a decrease of the E_33_ Young’s modulus and an increase of the G_13/23_ shear modulus. This compensation could also explain the difference between the updated moduli of this study (E_33_ = 12.88 GPa, G_13/23_ = 4.53 MPa) compared to the results of [7] (E_33_ = 13.90 GPa, G_13/23_ = 3.93 MPa), which studied different geometries of composite long bone models consisting of the same cortical and trabecular bone analogue material. An improved FE model, including an axis of transverse isotropy following the clavicle curvature, can be developed during future research to endorse this finding. Despite this assumption, it needs to be stated that the presented updated FE model accurately represents the experimental modal behavior and can be used for further in silico studies.

## 5. Conclusions

This study provides a composite clavicle FE model of which the anisotropic elastic material constants were updated using experimental modal analysis. A high level of agreement was obtained between both the experimental and FE model; the differences between the first five experimental and FE-predicted natural frequencies were minimized to 0.49% on average with a maximum of 1.73%. As FE simulations form a very useful, cost and relatively time efficient analysis tool, this updated model can find its application in future numerical studies that focus on the mechanical behavior of fourth-generation clavicle composite bone models.

## Figures and Tables

**Table 1:** Initial material properties of the studied composite bone model [33,34].

Cancellous	E [MPa]	ν	ρ [g/cc]			
Isotropic	155	0.30	0.27			
Cortical	E_11_ = E_22_ [GPa]	E_33_ [GPa]	G_13_ = G_23_ [GPa]	ν_12_	ν_23_	ρ [g/cc]
Transversely isotropic	10	16	3.3	0.26	0.26	1.64

**Table 2:** Sensitivities of the natural frequencies per change of the material properties, expressed in percentage change in frequency per percentage change in material property.

Sensitivities (%/%)	Cancellous		Cortical				
Mode Number	ν	E	ν_12_	ν_23_	E_11_ = E_22_	E_33_	G_13_ = G_23_
1	0.00	0.01	0.02	−0.01	0.01	0.36	0.10
2	0.00	0.01	0.01	−0.01	0.01	0.34	0.12
3	0.00	0.01	0.00	0.01	0.03	0.05	0.38
4	0.00	0.01	0.01	0.00	0.01	0.28	0.17
5	0.00	0.02	0.01	−0.01	0.02	0.28	0.16

**Table 3:** Overview of the experimental and numerical natural frequencies and the differences before and after updating the material properties of the numerical finite element (FE) model.

Mode Number	1	2	3	4	5
Experimental natural frequency [Hz]	818	1214	2221	2758	3337
Numerical (FE) frequency before updating [Hz]	872	1284	2008	2801	3372
*Difference before updating [%]*	6.6	5.7	−9.6	1.5	1.1
Numerical (FE) frequency after updating [Hz]	821	1217	2221	2761	3279
*Difference after updating [%]*	0.4	0.3	0.0	0.1	−1.7

**Table 4:** Overview of the updated material properties of the numerical FE model.

	Before Updating	After Updating
ρ_cort_ [g/cc]	1.640	1.658
E_33_ [GPa]	16.00	12.88
G_13_ = G_23_ [MPa]	3.30	4.53
